# Editorial: Construction of transcriptional and alternative splicing regulatory networks

**DOI:** 10.3389/fmolb.2023.1334202

**Published:** 2023-11-22

**Authors:** Wenbin Guo, Cristiane Paula Gomes Calixto, Zongtao Sun

**Affiliations:** ^1^ The James Hutton Institute, Dundee, United Kingdom; ^2^ University of São Paulo, São Paulo, Brazil; ^3^ Ningbo University, Ningbo, China

**Keywords:** gene regulatory network, alternative splicing regulatory network, high-throughput sequencing, RNA-seq, splicing factors, network inference techniques

Gene expression is a complex process, involving intricate layers of control, such as transcriptional and alternative splicing regulation. Recent advances in high-throughput sequencing technologies enable the studies of gene regulation networks at the transcript resolution, yielding an in-depth understanding of the intricate regulatory systems. This Research Topic focuses on the exploration and application of innovative network inference methods aimed at untangling the complexities of transcriptional and alternative splicing regulation.

The collected articles cover a wide range of topics. Some of the articles focus on the development of methods for network inference, such as the use of transcript and gene-level quantifications to infer regulatory networks. Other articles focus on the construction of alternative splicing regulatory networks from experimental data, as well as the cross-talk between transcription and alternative splicing networks. The contributions within this Research Topic explore strategies to integrate diverse biological data types, unveiling the mechanisms governing transcription and alternative splicing regulation through a wide spectrum of studies with human, animal, and plant data.

Different gene regulatory network technologies were used in human disease studies in this Research Topic. Zheng et al. built a microRNA-based network to uncover the molecular basis of neuropathic pain (NP) and its transcriptional regulation. The study scrutinised four electronic databases to identify commonly dysregulated miRNAs associated with NP. Through in-depth GO and KEGG pathway analyses, the research unveiled the functional specificity of differentially expressed miRNAs linked to pain. The results also spotlighted numerous miRNAs and their target genes entwined with well-established pathways related to NP. This discovery introduced promising possibilities for pain relief by targeting transient receptor potential channels, voltage-gated sodium channels, and voltage-gated calcium channels. Wang et al. studied the multifaceted regulatory mechanisms of DNA- and RNA-binding proteins (DRBPs) in chronic myeloid leukemia (CML). The study integrated and analysed ChIP-seq, CLIP-seq, RNA-seq and shRNA-seq data from the K562 cell line. This research uncovered a complex two-layer regulatory network system centred around four DRBP-SFs (DNA- and RNA-binding protein-splicing factors), and introduced three potential models in which DRBP-SFs cooperatively connect transcriptional and alternative splicing regulatory networks in CML. Their work opened fresh perspectives for the exploration of DRBPs in regulatory networks, offering potential insights into the intricate gene regulatory system central to CML.

In animal studies, He et al. investigated the impact of altering day length on the transcriptome of the hypothalamus. Establishing a comprehensive animal model using ovariectomised and estradiol-treated sheep, the study examined the plasma levels of pivotal reproductive hormones, including follicle-stimulating hormone (FSH) and prolactin, under varying photoperiod conditions using radioimmunoassay. The study unveiled differentially expressed genes and pathways in the hypothalamus, providing insights into the intricate molecular mechanisms governing photoperiod-induced regulation of reproductive activities, not only in sheep but also in diverse mammals. Zhang et al. investigated the role of *fgf9* and *rspo1* genes in the sex differentiation of Chinese giant salamanders. It revealed a noteworthy antagonistic relationship between these genes, showing significant variations in *fgf9* and *rspo1* expression levels between testes and ovaries, highlighting their influence during gonadal development.

This Research Topic encompasses two articles dedicated to plant studies. Liu et al. explored the molecular processes behind sex differentiation in mulberry flowers. The study compared gene expression and metabolomics between staminate flowers and pistillate flowers through integrated transcriptome and metabolome analyses. The findings presented differentially expressed genes and metabolites engaged in diverse pathways, including flavonoid biosynthesis, galactose metabolism, plant-pathogen interaction, and starch and sucrose metabolism. This insight revealed the intricate regulation of mulberry sex differentiation, offering prospects for better control over mulberry flower development. Boulanger et al. investigated the putative role of splicing factors (SFs) in rice’s basal thermotolerance. Their approach was to build a transcriptional co-expression network from a publicly available RNA-seq dataset of heat-stressed plants over a time course. The authors identified a number of SFs whose expression was correlated with alternatively spliced transcripts of key thermotolerance-related genes. This insight sheds light on the molecular mechanisms underpinning alternative splicing regulation in response to heat stress, with potential implications for developing innovative strategies to enhance rice yield in the face of a changing climate.

The discoveries within this Research Topic provide invaluable insights into the construction of transcriptional and alternative splicing regulatory networks. The development of network inference methods, coupled with the integration of diverse biological data types, contributes to the identification of pivotal regulatory nodes and pathways. This knowledge is instrumental in the development of innovative therapeutic strategies, and in finding solutions for better animal and plant breeding. The continued evolution of novel network inference techniques and the integration of an array of biological data types are fundamental in the identification of crucial regulatory nodes and pathways. We hope that this Research Topic will inspire further research in this exciting field.

